# Improved assembly of the *Pungitius pungitius* reference genome

**DOI:** 10.1093/g3journal/jkae126

**Published:** 2024-06-11

**Authors:** Dandan Wang, Pasi Rastas, Xueling Yi, Ari Löytynoja, Mikko Kivikoski, Xueyun Feng, Kerry Reid, Juha Merilä

**Affiliations:** Area of Ecology and Biodiversity, School of Biological Sciences, The University of Hong Kong, 999077, Hong Kong SAR; Institute of Biotechnology, University of Helsinki, Helsinki FI-00014, Finland; Area of Ecology and Biodiversity, School of Biological Sciences, The University of Hong Kong, 999077, Hong Kong SAR; Institute of Biotechnology, University of Helsinki, Helsinki FI-00014, Finland; Organismal and Evolutionary Biology Research Programme, Faculty of Biological and Environmental Sciences, University of Helsinki, Helsinki FI-00014, Finland; Ecological Genetics Research Unit, Organismal and Evolutionary Biology Research Programme, Faculty of Biological and Environmental Sciences, University of Helsinki, Helsinki FI-00014, Finland; Department of Computer Science, University of Helsinki, Helsinki FI-00014, Finland; Institute of Biotechnology, University of Helsinki, Helsinki FI-00014, Finland; Ecological Genetics Research Unit, Organismal and Evolutionary Biology Research Programme, Faculty of Biological and Environmental Sciences, University of Helsinki, Helsinki FI-00014, Finland; Area of Ecology and Biodiversity, School of Biological Sciences, The University of Hong Kong, 999077, Hong Kong SAR; Area of Ecology and Biodiversity, School of Biological Sciences, The University of Hong Kong, 999077, Hong Kong SAR; Ecological Genetics Research Unit, Organismal and Evolutionary Biology Research Programme, Faculty of Biological and Environmental Sciences, University of Helsinki, Helsinki FI-00014, Finland

**Keywords:** *Gasterosteus aculeatus*, genome assembly, nine-spined stickleback, repetitive elements, sex chromosomes

## Abstract

The nine-spined stickleback (*Pungitius pungitius*) has been increasingly used as a model system in studies of local adaptation and sex chromosome evolution but its current reference genome assembly is far from perfect, lacking distinct sex chromosomes. We generated an improved assembly of the nine-spined stickleback reference genome (98.3% BUSCO completeness) with the aid of linked-read mapping. While the new assembly (v8) was of similar size as the earlier version (v7), we were able to assign 4.4 times more contigs to the linkage groups and improve the contiguity of the genome. Moreover, the new assembly contains a ∼22.8 Mb Y-linked scaffold (LG22) consisting mainly of previously assigned X-contigs, putative Y-contigs, putative centromere contigs, and highly repetitive elements. The male individual showed an even mapping depth on LG12 (pseudo X chromosome) and LG22 (Y-linked scaffold) in the segregating sites, suggesting near-pure X and Y representation in the v8 assembly. A total of 26,803 genes were annotated, and about 33% of the assembly was found to consist of repetitive elements. The high proportion of repetitive elements in LG22 (53.10%) suggests it can be difficult to assemble the complete sequence of the species’ Y chromosome. Nevertheless, the new assembly is a significant improvement over the previous version and should provide a valuable resource for genomic studies of stickleback fishes.

## Introduction

Reference genome assemblies are important resources for population genomic studies allowing researchers to collect high-quality variant data and identify the genetic basis of phenotypes of interest (Ellegren 2014; Thorburn *et al*. 2023). However, assemblies are seldom, if ever, complete representations of a given species’ genome both because of intraspecies diversity and technical challenges in genome assembly (Sherman *et al*. 2020; Kivikoski *et al*. 2021; Prasad *et al*. 2022). Sex chromosomes can be especially tricky to assemble because of the complex nature of sex determination regions and different sequence components of homologous sex chromosomes (Fraser *et al*. 2020; Carey *et al*. 2022), particularly on the sex-limited chromosomes (i.e. Y and W; Chang and Larracuente 2017; Tomaszkiewicz *et al*. 2017). As a result, highly repetitive and heterochromatic Y and W chromosomes are lacking from many existing reference assemblies. However, full assemblies of both sex chromosomes are important for the study of sex chromosome evolution (Peichel *et al*. 2020), sexually antagonistic selection (Bissegger *et al*. 2020), and speciation (Sætre *et al*. 2003; Presgraves 2008).

The stickleback fishes in the genus *Pungitius* have been subject to a lot of recent population genomic research focusing on the evolution of sex chromosomes (Shikano *et al*. 2011; Natri *et al*. 2013, 2019; Dixon *et al*. 2019), hybridization (Feng *et al*. 2022, 2024; Wang *et al*. 2023), adaptation (Herczeg *et al*. 2009; Shapiro *et al*. 2009; Fang *et al*. 2021; Kemppainen *et al*. 2021), and phylogeography (Aldenhoven *et al*. 2010; Guo *et al*. 2019; Feng *et al*. 2022; Wang *et al*. 2022). As is the case of many other taxa, the research in this model system has quickly evolved from usage of microsatellites (Aldenhoven *et al*. 2010; Shikano *et al*. 2010), mitochondrial DNA (Teacher *et al*. 2012) and restriction-site associated DNA sequencing (Bruneaux *et al*. 2013; Guo *et al*. 2019) to whole-genome resequencing (WGR, Kivikoski *et al*. 2021; Feng *et al*. 2023, 2024). However, the effective utilization of WGR data requires a high-quality reference genome to avoid biases and errors in downstream analyses.

The first publicly available reference genome assembly (v6) for the nine-spined stickleback (*Pungitius pungitius*) was published in 2019 (Varadharajan *et al*. 2019). While the quality of this assembly at the time of its publication was considered to be high, a re-assembly (v7) 2 years later further improved its contiguity (Kivikoski *et al*. 2021). However, even the latter assembly could not fully resolve the sex chromosomes: the linkage group for the X chromosome (LG12) contains incorrectly polarized regions and the Y chromosome was left completely unassembled (Kivikoski *et al*. 2021). The aim of this study was to further improve the quality and contiguity of the nine-spined stickleback genome and in particular, attempt to assemble the two sex chromosomes with the aid of linked-read sequencing. The results show that the reassembly led to significant improvements of the reference genome overall with likely positive effects on read mapping and inferences on variants called from the nine-spined stickleback reference genome assembly, especially for the sex chromosomes represented by separate linkage groups (LGs).

## Materials and methods

### Genome assembly

The v8 reference genome was assembled from the complete set of contigs generated using PacBio sequencing (the same contigs as in the v6 and v7 assemblies; Varadharajan *et al*. 2019; Kivikoski *et al*. 2021). To improve the assembly, we generated linkage maps and genomic proximity by incorporating genomic information from additional alignment data. To obtain more accurate linkage maps, we sequenced two additional wild-caught individuals (one per sex) from the v7 reference population (FIN-PYO, 66.26226°N, 29.42916°E) using 10X Genomics linked-read sequencing (Elyanow *et al*. 2018; Nath *et al*. 2021) at ∼100 × coverage. Raw reads of 10X Genomics sequencing conducted by Novogene Europe were mapped to the contig-level assembly (available from v6; Varadharajan *et al*. 2019) using BWA-MEM (Li 2013). Then female alignments and male alignments were merged into two separate bam files and sorted using SAMtools (Li *et al*. 2009). The unique molecular identifier (UMI) barcodes of linked-reads were identified and marked in the merged alignments. Mapping depth (i.e. the number of unique UMIs) of each genomic region was calculated and used to classify these regions with the modules CoverageAnalyser and CoverageHMM in Lep-Anchor (Rastas 2020). These modules classify genomic regions automatically by fitting two Gaussian distributions with means of 50%, 100% and a zeta distribution (about 0% or over 100%) to the mapping depth. Because the whole contig sets consist of haploid and diploid contigs, the genomic regions were classified into three types based on the coverage of the male and female 10X Genomics data. The genomic regions were classified as “autosome” or “haplotype” (which will be removed from the contig sets) if both sexes had 100% or 50% of coverage; “X chromosome” if 50% of coverage in males and 100% in females; “Y chromosome” if 50% of coverage in males and 0% in females. We then combined the ultra-high-density linkage maps (Kivikoski *et al*. 2021) with the clustered genomic X and Y regions. The Y regions were added as markers in a new linkage group (LG22) and X regions were added as markers to the linkage group LG12. The pseudoautosomal region (PAR) of the sex chromosomes was separated from the regions in which both male and female had 100% coverage and kept in the LG12. These new markers formed an additional linkage map with only one map position (0cM) for LG12 and LG22. The final linkage maps (available from https://github.com/dandanWang2019/Pungitius_v8.git) were used in the genome improvement.

To further improve the genome assembly, we incorporated additional genomic proximity and alignment data from three additional sources: (1) To obtain proximity information for genomic regions, we mapped the 10X Genomics sequencing data to the genome and calculated mapping depth per 10 kb region in each contig. The mapping depths were variable across genomic regions and were thus normalized utilizing an approach used for Hi-C data (Lyu *et al*. 2020), with three iterative rounds which were sufficient to reach convergence. If the mapping depth dropped below 30 at a position between two regions both having depth over 30, then the contig was split into two regions by this position and the two regions could be nonadjacent in the assembly. (2) We generated contig-contig alignment chains using minimap2 (Li 2018) and scripts provided with Lep-Anchor. Additionally, the same alignment chain as in v7, generated by HaploMerger2 (Huang *et al.* 2017), was used as well. Alignment chains were used to remove haplotypic regions that included both allelic contigs. (3) We mapped the raw PacBio reads of the reference individual to the generated contig assembly as was done in v7 assembly (Kivikoski *et al*. 2021).

The above-generated linkage maps, genomic proximity, and contig-contig alignment chains were provided to Lep-Anchor (Rastas 2020) together with the alignment of the raw PacBio reads of the reference individual (available from Varadharajan *et al*. 2019). Lep-Anchor analyses were performed following the pipeline (https://sourceforge.net/p/lep-anchor/wiki/Home) and implemented in the wrapper lepanchor_wrapper3.sh. The LG19 inversion region was manually corrected in the v8 genome as in the v7 (Kivikoski *et al*. 2021).

The anchored v8 assembly contained 22 linkage groups, including a part of the putative Y chromosome assembled as LG22. We further refined LG22 by aligning it to the newly assembled LG12 (the pseudo X chromosome) using LAST (Frith *et al*. 2010). An artificial map was generated based on the alignments and all the contigs of LG22 were reordered based on the alignment using the Lep-Anchor module PlaceAndOrientContigs. Therefore, the order and orientation of contigs in nonrecombining LG22 does not necessarily reflect the reality, but in lack of linkage information for Y, no further refinement was possible.

We evaluated the completeness of the v8 assembly using BUSCO (Seppey *et al*. 2019) based on the actinopterygii_odb10 lineage dataset. The BUSCO analysis was performed without LG22 which contains regions homologous to the sex-linked region (SLR) of LG12. The gaps of autosomal sequences were calculated by assembly-stats (https://github.com/sanger-pathogens/assembly-stats).

### Identification of noncoding RNA genes

Noncoding RNA genes, including transfer RNA (tRNA) genes, ribosomal RNA (rRNA) genes, microRNA, and small nuclear RNA genes were identified. The tRNAscan-SE (v1.3.1; Schattner *et al*. 2005) was utilized to predict tRNA genes with eukaryote parameters. The rRNA genes were identified by RNAmmer (v1.2; Lagesen *et al*. 2007) with default parameters. The miRNA and snRNA genes were predicted by the homologous searching of the Rfam database using INFERNAL (v1.0.2; Nawrocki *et al*. 2009).

### Annotation of repetitive elements and genome structure

Repetitive elements, including tandem repeats and transposable elements, were identified in the v8 genome assembly. Tandem repeats were first annotated using Tandem Repeats Finder (TRF, v4.10.0; Benson 1999). Transposable elements were identified at both DNA and protein levels by combining *ab initio* and homology-based approaches. At the DNA level, RepeatMasker (v4.0.7; Chen 2004) “-species Actinopterygii” was used to search for similar transposable elements based on the known Repbase database (v20181026; Jurka *et al*. 2005). RepeatModeler (v1.0.11) within the RepeatMasker package was utilized to build a *de novo* repeat database of this assembly, which comprised a repeat consensus database with classification information, and RepeatMasker was used then to identify transposable elements using the *de novo* repeat database. At the protein level, RepeatProteinMasker (Chen 2004) within the RepeatMasker package was used to further search against the transposable element protein database using the WU-BLASTX engine. LTR_Finder (v1.06; Xu and Wang 2007) was applied to predict long terminal repeats (LTRs). The repeat sequences were masked before genome annotation.

To provide a comparison of repetitive element abundance with the closely related three-spined stickleback (*Gasterosteus aculeatus*) we repeated the analyses outlined above using the v5 three-spined stickleback reference genome based on a benthic male from Paxton Lake, Canada (Nath *et al*. 2021).

We identified the protein-coding genes through a comprehensive strategy with a combination of *de novo* prediction, homology-based and transcript-based prediction using the repeat-masked genome. For *de novo* prediction, we performed AUGUSTUS (v3.3.3; Stanke *et al*. 2006), GlimmerHMM (v3.0.4; Majoros *et al*. 2004), and GENSCAN (Burge and Karlin 1997). For homology-based prediction, GeMoMa (v1.6.4; Keilwagen *et al*. 2016) was used with default parameters and the published genomes from NCBI, including one model Teleostei (*Danio rerio*), and four related species (*G. aculeatus*, *Oreochromis niloticus*, *Oryzias latipes*, and *Xiphophorus maculatus*). We used each of these species as reference for homology-based prediction. For transcript-based prediction, we assembled five nine-spined stickleback transcriptomes from previous research (Wang *et al*. 2020) with the v8 genome assembly using StringTie (Pertea *et al*. 2015) and predicted its coding regions with TransDecoder (https://github.com/TransDecoder). Finally, all the gene annotation results were integrated with EvidenceModeler (EVM, v1.1.1; Haas *et al*. 2008), which generated a nonredundant consensus gene set. Then we assessed the completeness of the gene set using BUSCO (Seppey *et al*. 2019) based on the actinopterygii_odb10 lineage dataset.

### Realignment

To see how the new genome assembly improves mapping rate and sequence coverage of different genomic regions, as well as estimates of population genetic parameters such as nucleotide diversity, we utilized paired-end WGR data from six different nine-spined stickleback populations (31 males and 45 females; a total of 76 individuals) from previous research (Feng *et al*. 2022; Supplementary Table 1). The AdapterRemoval software (v2.3.1; Schubert *et al*. 2016) was used to remove the adapters and trim reads with both Ns and low-quality bases (-qualitymax 93). The overlapping reads (11 nucleotides, per default) were merged. Cleaned reads were aligned to the v7 and v8 reference genomes by BWA (v0.7.17) with the MEM algorithm, respectively. Female reads which mapped on LG12 and LG22 in v8 reference were extracted and remapped onto LG12. BAM files were then merged and sorted by SAMtools and duplicated reads were marked by the SAMtools markdup command. After alignment, the percentage of mapped reads was calculated by SAMtools (v1.16.1) flagstat function. The sequence coverage representing the proportion of the genomic region that was covered by reads, was calculated by SAMtools depth and Perl scripts. It was expressed as $\mathrm{coverage}=\frac{N}{G}$, where *N* is the total number of covered sites in the specific genomic regions of the reference genome, and *G* is the total length of the genomic regions. SAMtools script fixmate was used to fill in the mate-read positions.

### Variant calling

We called variants for 76 individuals using the v7 and v8 reference genomes, respectively. Variant calling for each individual was carried out separately by GATK (v4.3.0.0) HaplotypeCaller to produce GVCF files. Because of the putative Y chromosome assembled in v8, males showed different ploidies for sex chromosomes and autosomes. We called variants of males by setting -ploidy argument as 1 in v8 LG12 SLR (1–17,767,635 bp, see *Results*) and LG22. All GVCFs were then merged into 21 (22 in v8) single-chromosome GVCF files and a contig GVCF file with all unassigned contigs using the CombineGVCFs function. GenotypeGVCFs was used then to jointly genotype all samples and convert GVCF to VCF format. Then all VCF files were merged into one VCF file through the GATK MergeVcfs function. SNPs and indels were extracted by SelectVariants command. After that, variants were initially filtered using VariantFiltration (for SNPs: –filter-expression “MQRankSum < −12.5 || FS > 60.0 || ReadPosRankSum < −8.0 || MQ < 40.0 || QD < 2.0”; for indels: “QD < 2.0 || FS > 200.0 || ReadPosRankSum < −20.0”). We further filtered the SNPs by VCFtools (v0.1.17) with the setting of –minGQ 20 –minQ 30 –min-meanDP 3 –max-meanDP 35 –maf 0.05 –max-missing 0.2 –exclude-bed repeat.bed. The variants that only occurred in the reference individual were then filtered by VCFtools. Repeat regions of v7 were identified using RepeatMasker (v4.0.7) based on the repeat database of v8 annotation.

### Population genetic statistics

We estimated nucleotide diversity (*π*) using 100 kb nonoverlapping windows along each chromosome for high-quality SNP datasets generated by VCFtools (v0.1.17). For autosomes, the estimator was expressed as the average value of autosomes. The *π* for LG12 SLR and PAR were calculated separately. The SLR and PAR were identified in the v8 based on the synteny between v7 and v8 according to the corresponding regions in v7 (Kivikoski *et al*. 2021). VCFtools –haploid (https://github.com/jydu/vcftools) was used to process SNPs within LG12 SLR and LG22 for males. The difference between *π* of different genomic regions of v8 and v7 for each of the six populations (Supplementary Table 1; FIN-HEL, FIN-PYO, RUS-KRU, RUS-MAS, SWE-KIR, SWE-NAV) was tested with the Wilcoxon signed rank (for non-normal distribution) and paired *t*-tests (for normal distribution). Results were considered significant at *P* < 0.05.

## Results

### Genome assembly and annotation

**Fig. 1.**
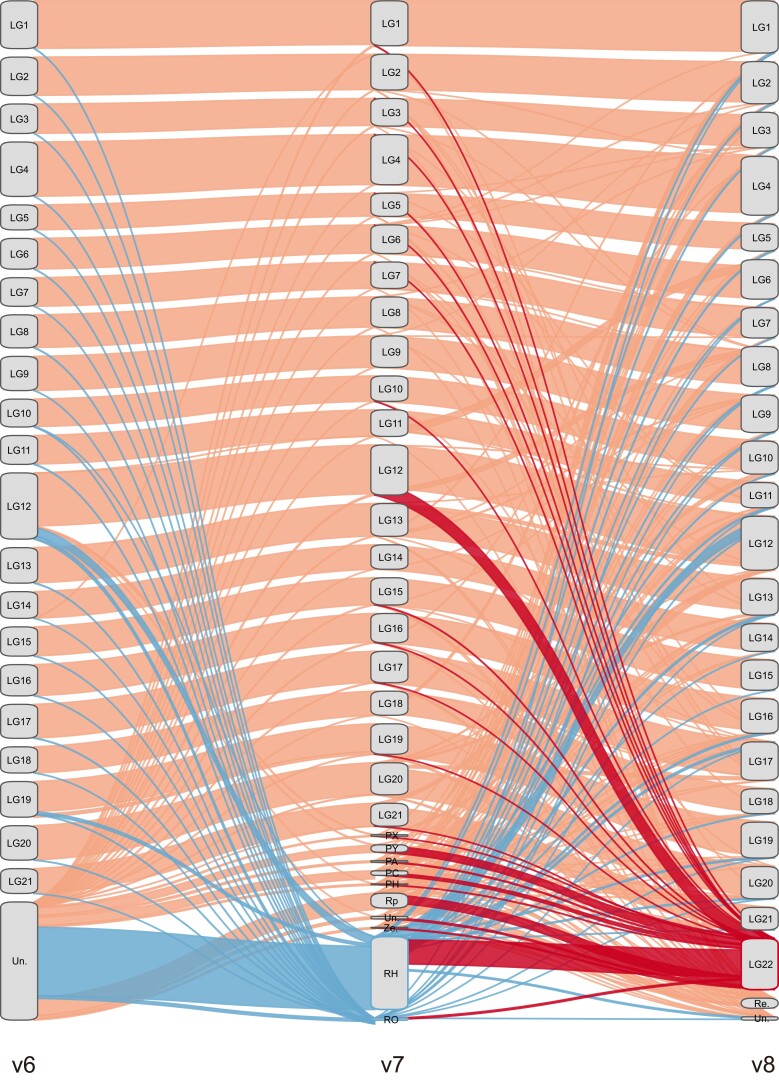
Changes in contig placement between v6, v7, and v8 nine-spined stickleback assemblies. Diagonal lines indicate changes in contig placement between different assemblies with band height corresponding to the length of the LGs. Certain contigs were rearranged to different LGs in v8. Red lines track the contigs assigned into LG22, and blue corresponds to the placement of the v7-removed contigs. PX, putative X contigs; PY, putative Y contigs; PA, putative autosome contigs; PC, putative centromere contigs; PH, putative haplotype contigs; Rp, repeat contigs; Un., unassigned contigs; Ze., zero coverage contigs; RO, v7-removed overlap contigs; RH, v7-removed haplotype contigs (note that the RO and RH contigs were excluded in the v7 reference genome); Re., removed contigs in v8.

We used the Lep-Anchor software (Rastas 2020), improved linkage maps, and 10X Genomics sequencing to reassemble the nine-spined stickleback genome. The new linked-read mapping allowed assigning 1,539 of the 1,644 previously unassigned contigs in v7 to different LGs, including a near-pure pseudo X chromosome (LG12) and a large Y-linked scaffold (LG22, ∼22.8 Mb; Fig. 1). Some v7-removed contigs (i.e. the contigs assembled in v6 but removed in v7, cf. Kivikoski *et al*. 2021) were assigned into the new assembly based on the coverage information (Fig. 1). Re-assignment of contigs led to an enhancement in genome contiguity, resulting in an increased N50 contig length of ∼1 Mb and the closure of 91 autosomal gaps (Table 1).

**Table 1.** Comparison of the three publicly available nine-spined stickleback genome assemblies.

Feature	v6	v7	v8	% change*^a^*
N50 scaffold size (bp)	1,202,809	19,652,119	20,475,552	+4.19
Total length of the assembly (bp)	521,233,387	466,582,808	466,451,302	−0.03
LG12 length (bp)	40,899,740	33,585,825	34,517,495	+2.77
Contigs in linkage groups	686	843	3,717	+340.93
Contigs in LG12	244	150	400	+166.67
Contigs not assigned to linkage groups (length)	4,616 (76,734,720 bp)	1,644 (27,251,636 bp)	105 (2,937,246 bp)	−93.61
Number of Gaps (Autosomes)	421	286	195	−31.81
Complete BUSCO	3,575 (98.2%)	3,575 (98.2%)	3,578 (98.3%)	+0.08
Complete single-copy BUSCOs	3,438 (94.5%)	3,530 (97%)	3,541 (97.3%)	+0.31
Complete duplicated BUSCOs	134 (3.7%)	45 (1.2%)	37 (1.0%)	−16.67
Fragmented BUSCOs	18 (0.5%)	14 (0.4%)	10 (0.3%)	−25
Missing BUSCOs	50 (1.3%)	51 (1.4%)	52 (1.4%)	0
Total BUSCO groups searched	3,640	3,640	3,640	—
Number of protein-coding genes	25,062	25,062	26,803	+6.95
Repeat sequence predicted (Mb)	121,030,392	121,030,392	153,161,593	+26.55

^*a*^Refers to the change from v7 to v8 assembly.

**Fig. 2.**
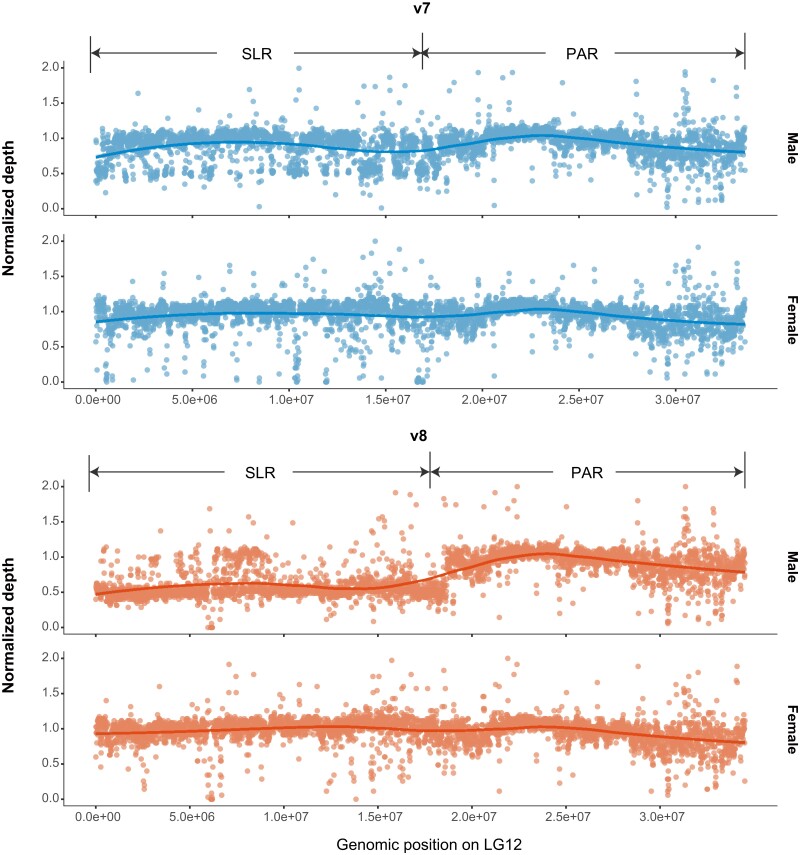
Improvements in assigning X-chromosome-linked contigs on LG12 pseudo-sex chromosome assembly. Normalized read depths of a male (top; FIN-HEL; 35X coverage) and a female (bottom; FIN-HEL; 35X coverage) are plotted. The solid lines show the trends of the normalized mapping depths along LG12 obtained with loess regression. The v7 assembly is a haploid representation with only one mosaic sequence copy (LG12) of homologous sex chromosomes and both sexes show even coverage over the full LG12. In v8, the male shows half of the coverage in the sex-linked region, because the complementary Y region is now on LG22 (Supplementary Fig. 1), and the female shows fewer regions with zero depth in v8 than in v7.

The v8 assembly improved the sex chromosomes (LG12 and LG22) in particular. The content of the LG12 changed considerably from v7 to v8 as previously LG12-assigned contigs (∼5.35 Mb) became incorporated into LG22, along with putative Y-contigs, putative centromere contigs and highly repetitive contigs. Once Y-linked contigs were reassembled to LG22, the remaining LG12 contigs (∼28.24 Mb) and some of the unassigned and v7-removed contigs were assembled in the new LG12 (Fig. 1), including putative X-contigs, putative centromere contigs and several highly repetitive contigs (Kivikoski *et al*. 2021). This resulted in a longer LG12 in v8 (∼34.52 Mb) compared to that in v7 (∼33.59 Mb; Supplementary Table 2). In addition, the SLR of LG12 was also longer in v8 (spanning 1–17,767,635 bp) compared to the consensus sequence in v7 (1–16,900,000 bp; Kivikoski *et al*. 2021). The other part of LG12 mainly represents the PAR which exhibited identical mapping depth in both male and females (Fig. 2). Importantly, the mapping analysis revealed that the male had half of the coverage of the female in the SLR of LG12 (Fig. 2), while LG22 showed minimal mapping for the female in the nonrepetitive parts (Supplementary Fig. 1). These findings provide further evidence for the near-pure X composition of LG12 SLR and the near-pure Y composition of LG22 in v8, contributing to the establishment of a more accurate haploid reference genome.

After reassigning contigs into linkage groups, we updated the annotation for the v8 assembly. Based on *ab initio*, homology-based, and transcript-based annotation, we predicted 26,803 genes, with an average gene length of 10,236 bp, an average coding DNA sequence (CDS) length of 1,533 bp, and on average nine exons per gene (Supplementary Table 3). BUSCO analysis of the protein-coding gene set showed that the annotated genome contained 92.5% Actinopterygii-conserved genes, suggesting high completeness and accuracy of protein-coding genes. The LG11 contained the highest proportion of genes (∼83.6/Mb), while the lowest gene density (∼44.7/Mb) was observed in LG22 (Supplementary Table 2). In addition, a total of 7,552 noncoding RNAs (ncRNAs) were also identified, including 1,278 ribosomal RNAs (rRNAs), 5,142 transfer RNAs (tRNAs), 481 microRNA (miRNAs), and 651 small nuclear RNAs (snRNAs; Supplementary Table 4).

We also detected 153.16 Mb (32.84%) repetitive elements in v8, which is ∼32 Mb more than in the v6 (v7 uses v6 annotation; Table 1). Transposable elements were predominant, accounting for 81.13% of all repeats (Table 2). Repeat sequence took up 53.10% of LG22, consisting of 13.83% tandem repeats, 11.47% LTRs, 14.7% DNAs (DNA transposons), and 10.89% LINEs (long interspersed nuclear elements), which suggests highly repetitive Y (Supplementary Fig. 2). Comparison to the re-annotated three-spined stickleback genome revealed that the two species have very similar repeat content across different repeat types including the total repeat content of the genome (Table 2).

**Table 2.** Classification of repetitive elements in the v6 and v8 assemblies of *P. pungitius* and re-annotation of *G. aculeatus* ver. 5.0.1 (Nath *et al*. 2021) repetitive elements for comparison.

	*Pungitius pungitius*				*Gasterosteus aculeatus*	
	v6		v8			
Type	Length (bp)	Proportion of genome (%)	Length (bp)	Proportion of genome (%)	Length (bp)	Proportion of genome (%)
DNA	36,017,227	6.91	64,096,778	13.741	69,885,312	14.800
DNA_CMC-EnSpm	—	—	5,643,084	1.210	6,964,626	1.475
DNA_MuDR	—	—	425,102	0.091	471,475	0.100
DNA_PIF-Harbinger	—	—	4,832,890	1.036	5,906,629	1.251
DNA_hAT-Ac	—	—	15,939,515	3.417	15,676,670	3.320
DNA_hAT-Tip100	—	—	5,503,795	1.180	5,323,319	1.127
DNA_Helitron	—	—	9,733,425	2.087	5,476,126	1.160
DNA_other	—	—	28,423,084	6.093	36,774,304	7.788
LINE	11,884,121	2.28	31,295,175	6.709	37,136,805	7.865
LINE_L1	—	—	4,230,937	0.907	3,597,545	0.762
LINE_L2	—	—	15,315,252	3.283	18,059,526	3.825
LINE_other	—	—	13,586,775	2.913	17,473,418	3.700
LTR	23,976,736	4.60	25,034,797	5.367	29,080,255	6.159
LTR_Copia	—	—	700,528	0.150	865,543	0.183
LTR_Gypsy	—	—	14,201,403	3.045	15,778,507	3.342
LTR_other	—	—	10,800,549	2.315	13,706,677	2.903
Low_complexity	—	—	1,336,220	0.286	1,124,589	0.238
SINE	2,606,167	0.50	3,843,603	0.824	4,284,677	0.907
Satellite	—	—	1,532,420	0.329	1,459,076	0.309
Simple_repeat	—	—	27,966,495	5.996	20,030,566	4.242
Small_RNA	—	—	1,510,277	0.324	532,134	0.113
**Total**	**121,030,392**	**23.22**	**153,161,593**	**32**.**840**	**154,729,762**	**32**.**770**

### Alignment to the reference genome

**Fig. 3.**
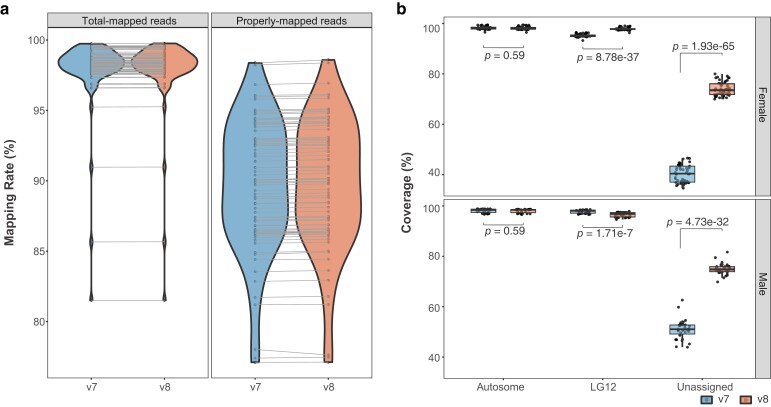
Mapping rate (a) and coverage (b) of 76 individuals when mapped on the v7 (blue) and v8 (red) assemblies, respectively. “Properly-mapped reads” in (a) refers to properly paired mapped reads, and “Total-mapped reads” to all mapped reads on the reference genome. The gray lines connect the values of the same individual in v7 and v8. Unassigned in (b) refers to unplaced contigs in corresponding genome assemblies. The comparisons between genomic regions of v7 and v8 were conducted by paired *t*-test.

To see if the v8 reassembly improves alignment quality, we mapped WGR data of 76 individuals from six populations (Supplementary Table 1) to both v7 and v8 assemblies and assessed the alignment quality based on the percent of mapped reads (mapping rates) and coverage. All individuals had higher mapping rates to the v8 genome (ranging from 81.17 to 99.75%, mean of 97.83%; Fig. 3a) compared to that of the v7 genome (ranging from 81.16 to 99.72%, mean of 97.81%; Fig. 3a). The percentage of properly paired mapped reads (both mates of a read pair are mapped close to each other in opposite directions on the same LG) was also higher in v8, ranging from 77.12 to 98.59% (mean of 89.69%), than in v7, ranging from 77.11 to 98.38% (mean of 89.62%; Fig. 3a). Two female individuals showed a minor (0.3 and 0.39%, respectively) decrease in properly paired reads when mapped to the v8 genome. There was no significant difference in autosomal coverage between the v8 and v7 reference genomes for both males and females (Fig. 3b and Supplementary Table 5). However, a significant increase and decrease of coverage in v8 LG12 were observed for females and males, respectively, in comparison to v7 LG12 (Fig. 3b and Supplementary Table 5), indicating a purer X composition of the v8 LG12 sex-linked region.

### Variant calling and population genetics

To investigate the difference in calling variants between reference genomes, we examined variants using v7 and v8 genomes, respectively. No big difference was observed in the number of variants in autosomes called using v8 reference genome (4,094,896; Supplementary Table 6) and v7 (4,041,744; Supplementary Table 6). However, fewer variants (both SNPs and indels) were found on the sex-linked region of v8 LG12 than that of v7 for both males and females (Supplementary Table 6).

As male reads mapped evenly on the LG12 SLR and LG22 (normalized depth around 0.5; Fig. 2 and Supplementary Fig. 1), we called variants of these regions using haploid mode for all males separately. A lower density of variants (males: ∼0.0074/bp, females: ∼0.0066/bp; Supplementary Table 6) was observed in the LG12 SLR than in the PAR (males: ∼0.0118/bp, females: ∼0.0106/bp; Supplementary Table 6). LG22 had the lowest variant density (∼0.0012/bp) among all LGs (∼0.0101/bp in autosomal LGs; Supplementary Table 6) as would be expected for nonrecombining Y chromosome.

**Fig. 4.**
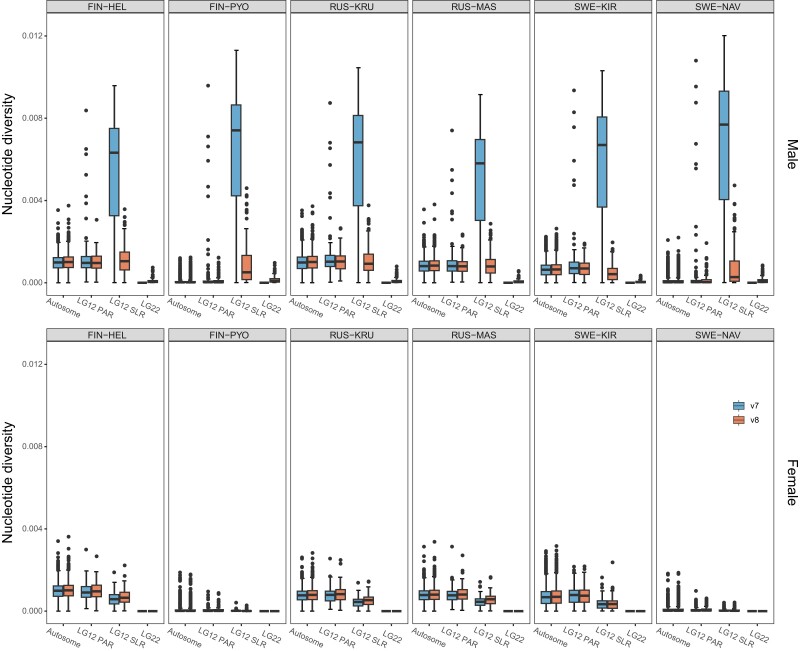
Nucleotide diversity in male and female nine-spined sticklebacks from six different populations over 100 kb nonoverlap windows based on autosomes, LG12 SLR, LG12 PAR, and LG22 of v7 and v8 reference genome. Note that female data mapped to LG22 (the Y-linked scaffold) were excluded from the analyses and the corresponding nucleotide diversity was zero in this plot.

To test the performance of the updated reference genome for population genetic inference, we compared nucleotide diversity (*π*) for different genomic regions of v7 and v8 reference genomes. Overall, no significant difference in *π* was observed between v7 and v8 in most genomic regions (autosomes and PAR; Fig. 4 and Supplementary Table 7; v7: mean = ∼0.00064, v8: mean = ∼0.00060; Wilcoxon signed rank test, *P* = 0.7257), except in the sex-linked region of LG12 for males (Fig. 4; v7: mean = ∼0.00576, v8: mean = ∼0.00085; paired *t*-test, *t_5_* = 17.505, *P* = 1.115e-05). Based on the v8 reference, the genetic diversity of LG12 SLR was lower than the $\frac{3}{4}$ neutral equilibrium expectation of that in autosomal LGs in females (Fig. 4; observed mean = ∼0.00036, expected mean = ∼0.00042; Wilcoxon signed rank test, *P* = 0.0313). Males had a higher *π* than females in LG12 SLR (Fig. 4 and Supplementary Table 7; v7: mean = ∼0.00576, v8: mean = ∼0.00085; paired *t*-test, *t_5_* = 4.476, *P* = 0.0065). The newly assembled LG22 showed the lowest *π* in all populations. In fact, *π* was lower than the one-fourth of autosomal expectation, except in the FIN-PYO and SWE-NAV populations where only two and three male individuals were available, respectively (Fig. 4, Supplementary Tables 1 and 7; observed mean = ∼0.00008, expected mean = ∼0.00021; paired *t*-test, *t_3_* = −12.433, *P* = 0.0011). The PAR exhibited significantly higher *π* than autosomes (Fig. 4 and Supplementary Table 7; PAR: mean = ∼0.00072, autosomes: mean = ∼0.00068; Wilcoxon signed rank test, *P* = 0.0020) in most comparisons, except for males from FIN-PYO and SWE-NAV.

## Discussion

The newly assembled reference genome is a clear improvement over the previously published v7 reference genome. Specifically, the updated version led to a significant decrease in unassigned contigs and moved many previously putatively mistakenly assigned Y-linked contigs to a new linkage group (LG22; sex-linked region of the species Y chromosome), leading to separated putative sex chromosomes. Furthermore, many of the earlier unassigned contigs identified as repetitive elements were moved to the putative Y chromosome, over 50% of which were found to consist of repetitive elements. Based on the new annotation, more genes and repetitive elements were predicted in the new assembly suggesting improved annotation over the v7 annotation which is a liftover from v6 reference genome. Furthermore, more contiguous LG sequences in v8 improved the quality of read mapping and for all individuals tested, the mappability of pair-end reads onto the v8 genome was increased. As such, the newly assembled v8 genome likely exhibits a more complete representation of the nine-spined stickleback genome than the earlier assemblies.

The higher quality of v8 was not only manifested in the improved contiguity of the autosomes but also in the separation of the sex chromosomes. The considerably changed LG12 and the newly assembled LG22 are suggested to represent the X and Y chromosomes, respectively. A clear depth differentiation between males and females was observed both in LG12 SLR and LG22. Because the PAR is kept in LG12 (17,767,635–34,517,495 bp), LG22 (∼22.8 Mb) appears to only consist of the sex-linked region of the nine-spined stickleback Y chromosome. Cytogenetic evidence has indicated that the nine-spined stickleback Y is larger than the X chromosome (Ocalewicz *et al*. 2008; Natri *et al*. 2019), and in line with that, the LG22 of the new v8 assembly is nearly five megabases longer than the homologous SLR in LG12 (∼22.8 vs ∼17.8 Mb, respectively). A higher repetitive sequence content was identified in LG22 compared to LG12 (Supplementary Fig. 2), which is typical for Y chromosomal SLR (Xue *et al*. 2021). Finally, we note that although 10X linked-read sequencing has contributed to genome refinements providing long-range information, 10X Genomics discontinued the technology in 2020. Further improvement of nine-spined stickleback sex chromosome assemblies could be obtained with the aid of high-fidelity (HiFi) long-reads produced through circular consensus sequencing as applied in human Y chromosome assembly (Rhie *et al*. 2023).

Fewer SNPs were called using v8 than v7 assembly on LG12 SLR in males, likely reflecting a purer X representation of LG12 SLR of the new assembly. Only reads from the X chromosomes can map onto this region with the v8 reference, which reduces the fixed X-Y difference in the SNPs. This suggests that v8 assembly provides an improved reference for population genomic analyses involving the variants in sex chromosomes (Fig. 4). At the same time, we observed that female reads can cover an average of ∼28% (21%–35%) of LG22 due to sequence similarity between LG12 SLR and LG22 (Supplementary Fig. 1). One consequence of this is that the application of the standard variant calling pipeline would yield a reduced number of variants to be called from v8 assembly for females, because nonuniquely mapped reads will be removed from variant calling. Hence, for population genetic analyses focused on sex chromosomes, the best solution would be to exclude LG22 when mapping females (or remap LG22 reads to LG12, as has been done in this study). As v8 separated SLR of X and Y into LG12 and LG22, the variants will reflect population differences rather than fixed differences between X and Y.

In demographic equilibrium, X and Y chromosomes are expected to have three-fourth and one-fourth of the nucleotide diversity of autosomes (Hill 1979; Caballero 1995; Wilson Sayres 2018). However, most nine-spined stickleback populations exhibited a significantly reduced nucleotide diversity in v8 LG12 SLR and LG22, with values notably lower than the neutral equilibrium expectations of three-fourth and one-fourth. This could be explained by the low recombination rate and tight sex linkage present in the sex-linked region of the X chromosomes. Since the X chromosome is only able to recombine in females, only two-third of X chromosomes undergo recombination in each generation. Consequently, the linkage disequilibrium would be greater on the X chromosomes than in autosomes and regions with a uniform genetic history would be larger (Schaffner 2004). The reduced recombination of SLR might contribute to the lower number of observed variants in the SLR compared to PAR. Additional explanations for this difference include fewer SLR sequences (compared to autosomes) being involved in variant identification and selection against recessive variants within SLR that are only exposed in males. LG22 manifested extremely low nucleotide diversity far below the one-fourth expectation. This may not only be a result of decreased recombination and strong linkage but could also be due to the impact of purifying selection on the harmful elements accumulating on nonrecombining Y chromosomes (Wilson Sayres *et al*. 2014). Further studies are needed to evaluate these possibilities.

Unexpectedly, a higher *π* was observed in males than females within LG12 SLR with both v8 and v7 references. In addition, more variants were identified in males than females within LG12 SLR. One possible explanation for these observations is that some Y-reads were mapped on the LG12 SLR. This inter-sex difference is much stronger in the v7 LG12 SLR where a choice was made to represent the SLR as a single copy and the Y-reads were forced to map to the mostly X-origin LG12. Having two copies of the SLR greatly reduces the *π* of males in the v8 LG12 SLR and indicates that the X and Y reads are correctly separated. A higher *π* value was still observed in LG12 PAR than in autosomes, possibly due to balancing selection (Qiu *et al*. 2013), higher recombination rates (Kawakami *et al*. 2014; Martin *et al*. 2016), and/or higher mutation rates (Avia *et al*. 2018) in the PAR. These possibilities need to be evaluated in future studies.

The new assembly contained significantly more repetitive elements than the v6 assembly (v6: 23.22%; v8: 32.84%). Although this is a high figure, it is not exceptional as many fish species are known to have an even higher content of repetitive elements (Yuan *et al*. 2018; Shao *et al*. 2019; Hotaling *et al.* 2023). In the same vein, while earlier analyses suggested that the nine-spined sticklebacks have higher repetitive element content than the closely related three-spined stickleback (Varadharajan *et al*. 2019), our reanalysis of the three-spined stickleback assembly shows that the earlier comparison likely underestimated the repetitive element content in the latter species. Nevertheless, compared to their respective genome sizes, the two stickleback fishes appear to have higher repetitive element contents than most other fish species analyzed by Yuan *et al*. (2018) or Shao *et al*. (2019). One possible explanation for this could be lower assembly quality in earlier work which can lead to the omission of some repetitive elements as exemplified by our reanalysis of three-spined stickleback repetitive elements. However, high repeat content is also expected in species and populations with low effective population sizes because of the reduced efficiency of purifying selection (Lynch and Conery 2003; see also Supplementary Fig. 2 in Varadharajan *et al*. 2019). Many freshwater populations of nine-spined sticklebacks, including the one where the assembly reported in this paper comes from, have low effective population sizes (Feng *et al*. 2023). On the other hand, effective population sizes for three-spined sticklebacks are typically higher than those of the nine-spined sticklebacks (Fang *et al*. 2021) suggesting factors other than those relating to effective population size likely explain the high repetitive element content in these species. Given the potential link between transposable element activity and environmental conditions in fish (Yuan *et al*. 2018; Shao *et al*. 2019; Hotaling *et al*. 2023), it is also possible that a high abundance of repeat elements in sticklebacks is related to their circumpolar distribution in cool temperate and arctic waters.

In conclusion, the new v8 assembly is a significant improvement over the previous v7 assembly, being a more contiguous and complete representation of the nine-spined stickleback genome with a new annotation. That said, the assembly is based largely on a single male individual from one particular locality and as such, it is likely to provide only a partial window to the structure and genomic composition of the nine-spined stickleback genome. A pangenome (e.g. Gong *et al*. 2023) based on multiple individuals from different populations would likely capture much more structural variation than is contained in the current assembly and should be a focus of future research. Nevertheless, the improved v8 assembly should be a useful resource for stickleback research and for fish comparative genomics research.

## Supplementary Material

jkae126_Supplementary_Data

## Data Availability

The raw sequencing data of 10X Genomics have been deposited in the NCBI databases under BioProject accession PRJNA1045347. The v8 genome assembly of *P. pungitius* has been deposited at ENA and figshare (GCA_902500615; figshare link: https://figshare.com/s/f81ebd5cb3a8074df3c2). The genome annotation is available on the figshare data repository under accession doi: 10.6084/m9.figshare.24629751. PacBio reads are available from Varadharajan *et al*. (2019) and the short-read sequencing data used for population genetic analyses from Feng *et al*. (2022). For the information on individuals used see Supplementary Table 1. The linkage maps used in the v8 assembly and code used in the v8 annotation can be accessed at GitHub (https://github.com/dandanWang2019/Pungitius_v8.git). Supplemental material available at G3 online.
