# Supplementary material for: Improved assembly of the Pungitius pungitius reference genome
Source: G3 (Bethesda). 2024 Jun 11;14(8):jkae126. doi: 10.1093/g3journal/jkae126 (PMC11304971; doi:10.1093/g3journal/jkae126)
Supplement: jkae126_Supplementary_Data [file jkae126_supplementary_data.pdf]

Supplementary Figures

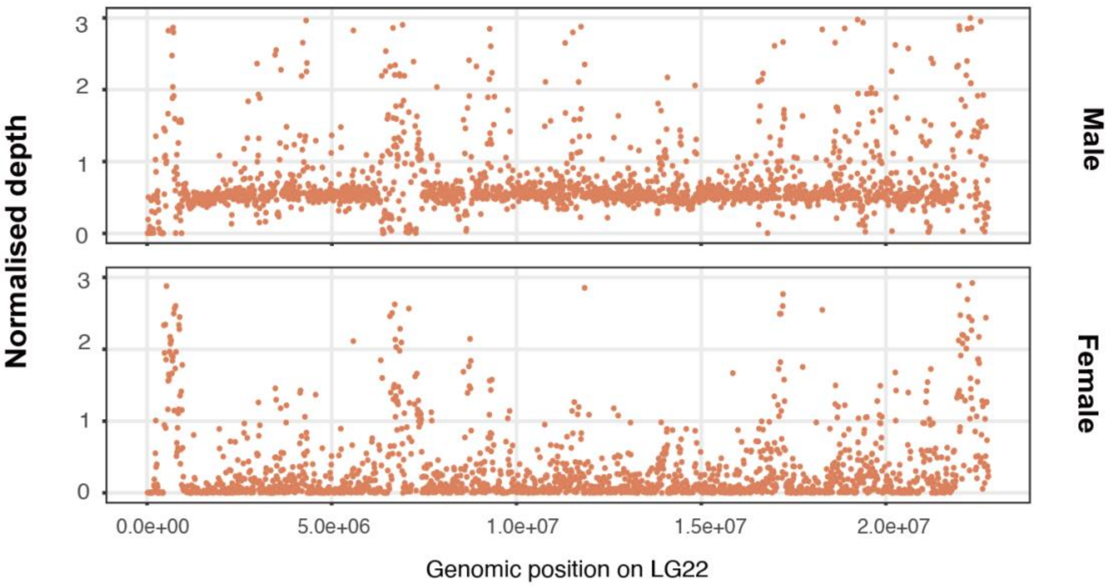

Figure S1 Normalised read depth on LG22. One male (35X coverage; top) and one female (35X coverage; bottom) from FIN-HEL population.

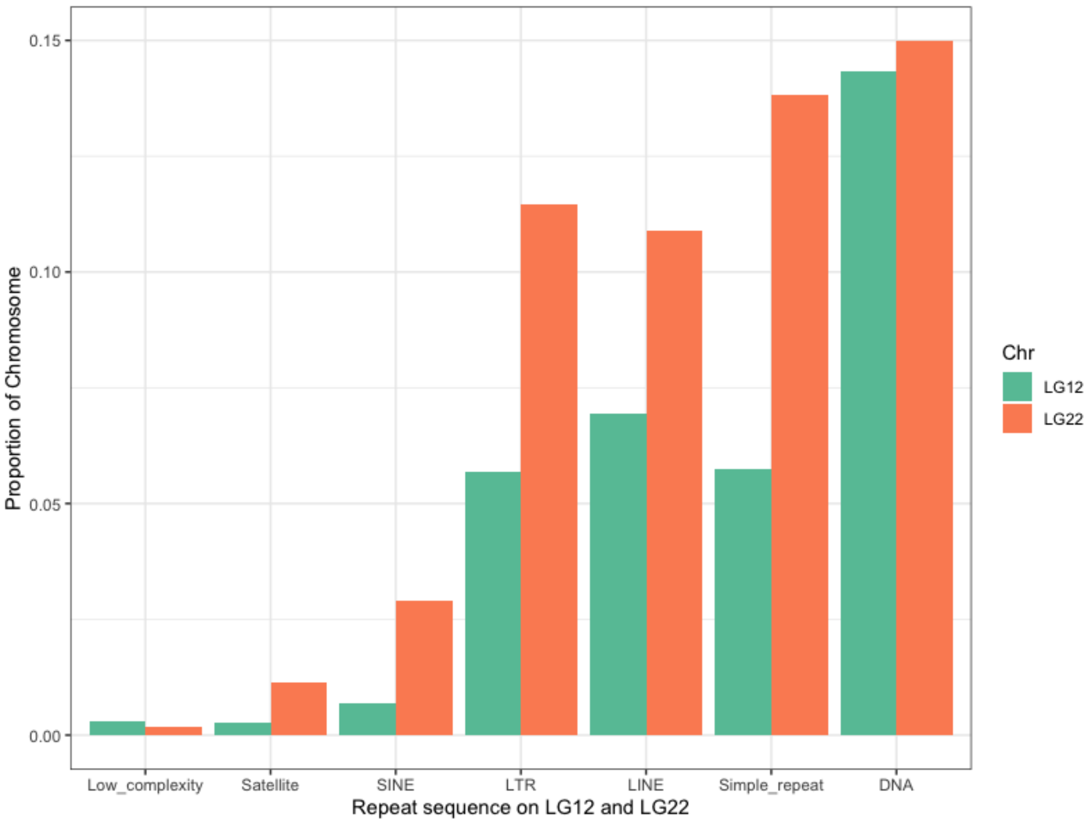

Figure S2 Repeat sequence percentage on LG12 and LG22. More repetitive elements occupied on LG22 (orange) than LG12 (green). Simple repeat and LTR categories show the largest difference.

Supplementary Tables

Table S1 Short-read sequencing data of 76 individuals used in population genetics analyses.

Accession ID	Sample ID	Sex	Population
ERR10006612	SWE-KIR-1	Female	SWE-KIR
ERR10006615	SWE-KIR-11	Male	SWE-KIR
ERR10006618	SWE-KIR-2	Female	SWE-KIR
ERR10006619	SWE-KIR-3	Male	SWE-KIR
ERR10006684	SWE-KIR-35	Male	SWE-KIR
ERR10006686	SWE-KIR-36	Male	SWE-KIR
ERR10006689	SWE-KIR-37	Female	SWE-KIR
ERR10006691	SWE-KIR-38	Male	SWE-KIR
ERR10006694	SWE-KIR-39	Male	SWE-KIR
ERR10006872	SWE-KIR-4	Male	SWE-KIR
ERR10006877	SWE-KIR-6	Male	SWE-KIR
ERR10006879	SWE-KIR-7	Male	SWE-KIR
ERR10006882	SWE-KIR-8	Male	SWE-KIR
ERR10006914	SWE-NAV-1	Female	SWE-NAV
ERR10006916	SWE-NAV-10	Female	SWE-NAV
ERR10006918	SWE-NAV-12	Female	SWE-NAV
ERR10006920	SWE-NAV-13	Female	SWE-NAV
ERR10006922	SWE-NAV-14	Male	SWE-NAV
ERR10006929	SWE-NAV-20	Female	SWE-NAV
ERR10006931	SWE-NAV-21	Female	SWE-NAV
ERR10006933	SWE-NAV-24	Female	SWE-NAV
ERR10006935	SWE-NAV-25	Female	SWE-NAV
ERR10006937	SWE-NAV-26	Male	SWE-NAV
ERR10006939	SWE-NAV-28	Female	SWE-NAV
ERR10006942	SWE-NAV-31	Male	SWE-NAV
ERR10006947	SWE-NAV-4	Female	SWE-NAV
ERR10048058	1-f	Female	FIN-HEL
ERR10048060	10-f	Female	FIN-HEL
ERR10048103	23-f	Female	FIN-HEL
ERR10048105	24-m-1	Male	FIN-HEL
ERR10048108	26-m-1	Male	FIN-HEL
ERR10048114	33-m-1	Male	FIN-HEL
ERR10048123	34-m-1	Male	FIN-HEL
ERR10048136	50-f	Female	FIN-HEL
ERR10048144	8-f	Female	FIN-HEL
ERR10048146	85-m-2	Male	FIN-HEL
ERR4408319	FIN-PYO-25	Male	FIN-PYO
ERR9993593	FIN-PYO-1	Female	FIN-PYO
ERR9993594	FIN-PYO-17	Female	FIN-PYO
ERR9993595	FIN-PYO-18	Female	FIN-PYO
ERR9993597	FIN-PYO-2	Male	FIN-PYO

ERR9993598	FIN-PYO-24	Female	FIN-PYO
ERR9993599	FIN-PYO-26	Female	FIN-PYO
ERR9993695	FIN-PYO-30	Female	FIN-PYO
ERR9993696	FIN-PYO-31	Female	FIN-PYO
ERR9993697	FIN-PYO-34	Female	FIN-PYO
ERR9993759	FIN-PYO-48	Female	FIN-PYO
ERR9993762	FIN-PYO-5	Female	FIN-PYO
ERR9994208	FIN-PYO-58	Female	FIN-PYO
ERR9994209	FIN-PYO-59	Female	FIN-PYO
ERR9998482	RUS-KRU-1	Female	RUS-KRU
ERR9998483	RUS-KRU-11	Female	RUS-KRU
ERR9998484	RUS-KRU-12	Female	RUS-KRU
ERR9998487	RUS-KRU-13	Female	RUS-KRU
ERR9998488	RUS-KRU-14	Female	RUS-KRU
ERR9998491	RUS-KRU-19	Female	RUS-KRU
ERR9998493	RUS-KRU-20	Female	RUS-KRU
ERR9998497	RUS-KRU-28	Male	RUS-KRU
ERR9998500	RUS-KRU-3	Female	RUS-KRU
ERR9998502	RUS-KRU-31	Male	RUS-KRU
ERR9998559	RUS-KRU-4	Male	RUS-KRU
ERR9998561	RUS-KRU-5	Male	RUS-KRU
ERR9998563	RUS-KRU-9	Male	RUS-KRU
ERR9999213	RUS-MAS-11	Female	RUS-MAS
ERR9999217	RUS-MAS-13	Female	RUS-MAS
ERR9999219	RUS-MAS-14	Female	RUS-MAS
ERR9999224	RUS-MAS-45	Male	RUS-MAS
ERR9999323	RUS-MAS-46	Male	RUS-MAS
ERR9999324	RUS-MAS-47	Male	RUS-MAS
ERR9999325	RUS-MAS-48	Male	RUS-MAS
ERR9999327	RUS-MAS-49	Male	RUS-MAS
ERR9999329	RUS-MAS-55	Male	RUS-MAS
ERR9999333	RUS-MAS-6	Female	RUS-MAS
ERR9999335	RUS-MAS-7	Female	RUS-MAS
ERR9999336	RUS-MAS-8	Female	RUS-MAS
ERR9999338	RUS-MAS-9	Female	RUS-MAS

Table S2 Linkage group length of *P. pungitius* v7 and v8 assemblies and number of genes identified in v8.

Chromosome	v7	v8	Number of genes	Number of genes/Mb ratio
LG1	30,106,087	30,230,421	1,560	51.60364786
LG2	24,029,954	23,791,474	1,112	46.73943279
LG3	18,520,836	18,712,928	1,156	61.77547415
LG4	33,625,550	33,979,705	1,732	50.97160202
LG5	15,538,166	15,449,788	907	58.70630717
LG6	19,164,645	18,929,699	1,080	57.05320512
LG7	17,739,606	17,670,445	1,073	60.72286238
LG8	20,480,644	20,475,552	1,145	55.92034833
LG9	21,145,378	21,207,684	1,292	60.92131512
LG10	17,012,775	17,148,797	1,086	63.32805736
LG11	17,852,859	17,925,936	1,499	83.62185383
LG12	33,585,825	34,517,495	2,714	78.62679491
LG13	21,992,815	22,103,899	1,245	56.32490449
LG14	16,408,376	16,409,882	907	55.27157356
LG15	18,287,517	17,856,078	1,070	59.92357336
LG16	19,652,119	19,466,964	996	51.16360209
LG17	21,092,886	21,400,467	1,229	57.42865331
LG18	16,178,624	16,099,618	878	54.53545544
LG19	20,450,314	20,548,867	1,235	60.10063718
LG20	21,492,179	21,426,341	1,132	52.83216579
LG21	15,364,080	15,339,073	743	48.43838999
LG22	-	22,782,145	1,018	44.68411557
putY	-	24,078	0	0

Table S3 Protein-coding genes in the *P. pungitius* reference genome.

Gene set		Total Genes Predicted	Average Gene Length (bp)	Average CDS Length (bp)	Average Exons per Gene	Average Exon Length bp)	Average Intron Length (bp)
	AUGUSTUS	26,156	9,301.56	1,460.29	8.69	168.08	1,019.67
De novo	GLIMMER	152,012	2,268.08	473.42	2.95	160.48	920.31
	GENSCAN	23,494	14,349.72	1,695.43	10.02	169.21	1,402.96
	D. rerio	20,288	8,346.01	1,692.75	10.02	168.98	737.80
	G. aculeatus	22,511	8,499.97	1,780.64	10.26	173.49	725.33
Homolog	*O. niloticus*	23,479	8,487.27	1,806.95	10.13	178.37	731.68
	O. latipes	21,797	8,708.46	1,827.02	10.38	175.96	733.40
	X. maculatus	21,762	8,921.26	1,834.59	10.53	174.15	743.26
Transcript		20,349	12,239.51	1,420.65	8.74	162.46	966.47
**Final Set**		26,803	10,236.56	1,532.97	9.32	164.50	1,046.21

Table S4 Non-coding RNA genes in the *P. pungitius* reference genome.

Type	Copy number	Average length (bp)	Total length (bp)	Proportion of genome (%)
miRNA	481	75.23	36,185	0.0078
rRNA	1,278	185.32	236,835	0.0508
snRNA	651	131.45	85,575	0.0183
tRNA	5,142	76.90	395,414	0.0848

Table S5 Average genome coverage per LG for mapping of 76 Individuals to v7 genome and v8 genome.

	Male		Female	
	**v7 (%)**	**v8 (%)**	**v7 (%)**	**v8 (%)**
LG1	98.40	98.08	98.48	98.25
LG2	98.33	98.60	98.41	98.69
LG3	97.14	96.63	97.54	97.09
LG4	98.06	97.90	98.19	98.00
LG5	97.68	97.73	97.84	97.91
LG6	98.31	98.50	98.44	98.66
LG7	98.51	98.49	98.65	98.63
LG8	98.28	98.09	98.46	98.26
LG9	98.22	98.19	98.37	98.34
LG10	98.20	97.90	98.24	98.16
LG11	97.81	97.62	97.99	97.84
LG12	97.79	96.60	95.22	97.87
LG13	97.81	97.62	97.94	97.71
LG14	98.59	98.56	98.82	98.79
LG15	98.01	98.40	98.01	98.53
LG16	98.08	97.44	98.16	97.62
LG17	98.58	98.10	98.61	98.20
LG18	98.14	98.22	98.33	98.43
LG19	98.43	98.12	98.52	98.16
LG20	97.74	97.84	97.94	98.05
LG21	97.97	98.17	98.19	98.39
LG22	-	89.31	-	27.69
Unassigned contigs	50.97	75.01	40.37	73.99

Table S6 Variants called by v7 and v8 assemblies.

		Number of SNPs	Number of indels	Number of SNPs	Number of indels	Number of variant/bp
		v7		**v8**		**v8**
	Contigs	9,956	26,396	1,881	6,360	-
	Autosomes	2,022,121	2,019,623	2,073,906	2,020,990	0.0101
males	LG12_SLR	230,130	144,924	79,047	52,151	0.0074
	LG12_PAR	96,325	111,294	91,344	106,434	0.0118
	LG12	326,455	256,218	170,391	158,585	-
	LG22	-	-	10,938	17,086	0.0012
females	LG12_SLR	227,940	143,345	56,027	61,454	0.0066
	LG12_PAR	96,534	111,212	89,681	88,167	0.0106
	LG12	324,474	254,557	145,708	149,621	-

Table S7 Nucleotide diversity value in six populations with different regions of v8 genome.

Population	Sex	Autosome	LG12_SDR	LG12_PAR	LG22
FIN-HEL	male	0.00097322	0.00109145	0.00099232	8.97E-05
FIN-HEL	female	0.00097597	0.000665	0.00098829	NA
FIN-PYO	male	5.99E-05	0.00091664	0.00011247	0.0001652
FIN-PYO	female	5.68E-05	1.84E-05	6.95E-05	NA
RUS-KRU	male	0.00098254	0.00103297	0.00103404	8.94E-05
RUS-KRU	female	0.00076506	0.00052086	0.00083213	NA
RUS-MAS	male	0.00081572	0.00085279	0.00082701	7.06E-05
RUS-MAS	female	0.00077924	0.00053997	0.00083655	NA
SWE-KIR	male	0.00065133	0.00050449	0.00072142	5.44E-05
SWE-KIR	female	0.00070785	0.00037127	0.00076571	NA
SWE-NAV	male	8.91E-05	0.00072426	0.00013254	0.0001246
SWE-NAV	female	8.04E-05	5.07E-05	8.64E-05	NA
